# Assessment of Bacterial Infections and Antibiotic Regimens in Intravenous Drug Users

**DOI:** 10.7759/cureus.45716

**Published:** 2023-09-21

**Authors:** Sana Rehman, Sehrish Arif, Lekshmi G Ushakumari, Jasiya Amreen, Akshaya Nagelli, Sania J Moonnumackel, Arun Nair

**Affiliations:** 1 Medicine, Fatima Memorial Hospital (FMH) College of Medicine and Dentistry, Lahore, PAK; 2 Medicine, Jagadguru Jayadeva Murugarajendra (JJM) Medical College, Davanagere, IND; 3 Medicine, Dr. Vizarath Rasool Khan (VRK) Women's Medical College, Hyderabad, IND; 4 Medicine, Siddhartha Medical College, Vijayawada, IND; 5 Internal Medicine, Medical City Fort Worth, Fort Worth, USA; 6 Pediatrics, Saint Peter’s University Hospital, Somerset, USA

**Keywords:** antibiotic resistance, bacterial infections, antibiotic stewardship, antibiotics, intravenous drug user

## Abstract

Bacterial infections in people who inject drugs (PWID) are a significant cause of hospitalizations and increased morbidity in this group. In this review, we evaluated bacterial trends in the most common infections and appropriate empiric antibiotic coverage. Articles from PubMed and Google Scholar were curated in a Google document with shared access. Discussion and development of the paper were achieved over Zoom meetings. The common infections in PWID were skin and soft tissue infections (SSTIs), infective endocarditis, septic arthritis, osteomyelitis, and bloodstream infections (BSIs). The presence of comorbidities increased susceptibility to bacterial infections. *Staphylococcus aureus* was the predominant species in all the infections and included methicillin-resistant *S. aureus* (MRSA) and methicillin-sensitive *S. aureus* (MSSA). The current standard of antibiotic use for *Staphylococcus* species was found to be sufficient. The gram-negative bacillus *Serratia marcescens* was prevalent in PWID as a causative agent of septic arthritis, osteomyelitis, and infective endocarditis. Its treatment required a combination of β-lactam and either a fluoroquinolone or an aminoglycoside for a prolonged duration. Streptococci were commonly implicated in SSTIs and BSIs. The appropriate response was seen with β-lactam antibiotics. In PWID, *Enterococcus* and *Pseudomonas* were implicated in infective endocarditis of native and prosthetic valves. The former being difficult to treat, a new strategy of using dual β-lactam antibiotics was found to be supported by clinical data. Anti-pseudomonal antibiotics can be avoided in other infections seen in this group, as their prevalence was low. PWID, especially those with comorbidities, have an increased risk of acquiring infections with difficult-to-treat microbes. Therefore, empiric antibiotic treatment should be relevant to the infection, for which bacterial trends and antibiotic susceptibility must be reassessed periodically.

## Introduction and background

According to a 2017 systematic review done to establish the prevalence of injection drug use (IDU), led by a global consortium, 179 countries in the world have evidence of IDU in the 15-64 year age group [1]. Data from 83 countries shows evidence of 15.6 million people who inject drugs (PWID), with 3.2 million women and 12.5 million men. A total of 83.9% of PWID inject chiefly opioids, while 33% inject chiefly stimulants, with a combination of the two reported in a few countries [1]. In addition, 25.3% of PWID globally are younger than 25 years, and this subset is more prone to engage in risky behaviors such as needle and syringe sharing and high-risk sexual behaviors [2,3]. Some commonly seen risk factors for intravenous drug users (IVDUs) are recent homelessness or unstable housing, history of incarceration, involvement with sex work, and recent high-risk sexual activities [1].

Bacterial infection is a common complication seen in PWID, with skin and soft tissue infection (SSTI) being the most common reason for hospitalization in this group. Heroin use, specifically black tar heroin and speedball (heroin and cocaine combined), is associated with higher skin infection rates. In addition to SSTI, bacteremia, community-acquired pneumonia, infective endocarditis (IE), skeletal infections like osteomyelitis, and subdural and epidural abscesses are a few other common infections seen in PWID [4]. These complications increase the rate of hospitalizations and hospital expenses, the development of severe comorbidities like kidney injury, septic emboli, and stroke, and increase long-term mortality among PWID [4-10]. This situation is especially grave considering that the escalated need for antibiotics among this group leads to the emergence of multidrug-resistant bacteria and methicillin-resistant *Staphylococcus aureus* (MRSA) colonization [6,11,12]. From the point of view of public health, PWID infected with these strains could introduce them to the general public or immunocompromised people [11]. Apart from drug resistance, antibiotics could unfavorably react with commonly injected drugs. For example, serotonin syndrome must be kept in mind when using linezolid in opioid users, and rifampin, a CYP3A inducer, could potentially induce withdrawal in methadone users [7]. Ultimately, due to the general stigmatization of PWID, they are often not treated effectively for the pain from the infections or for their withdrawals during hospitalizations, making them more likely to use illicit drugs in an effort to treat themselves for it [8,11].

In PWID, *S. aureus* is one of the most common organisms causing superficial skin infections, community-acquired pneumonia, infective endocarditis, osteomyelitis, septic arthritis, and bacteremia [13-18]. However, many of these conditions seen in PWID also have concomitant identification of other prominent bacteria. Group A Streptococci, too are common culprits in superficial skin infections with abscesses usually containing oral bacterial flora and anaerobes [16,17]. In addition to *Staphylococcus*, anaerobes are also implicated in community-acquired pneumonia in PWID [17]. Organisms like the HACEK group (*Haemophilus*, *Aggregatibacter*, *Cardiobacterium*, *Eikenella*, and *Kingella* species) and *Candida* species are other important causative agents of IE. Furthermore, *Serratia* spp. was found to cause up to 5.3% of cases of septic arthritis in PWID in a large study [19-21].

Early initiation of appropriate antibiotics, typically broad-spectrum, should help reduce many of the complications discussed above. Nevertheless, bacterial trends may change over time. For example, *Pseudomonas aeruginosa*, once a common cause of septic arthritis in PWID [19], is less common now [22]. Therefore, we conducted a literature review on the prominent bacteria seen in commonly occurring infections among PWID and the antibiotic susceptibility in each group. This should help formulate the best empiric antibiotic treatment and improve many undesirable parameters associated with a bacterial infection in PWID.

## Review

Methods

The design of this paper was decided to be a literature review in order to build a comprehensive view of our research topic and formulate conclusions and perspectives from existing knowledge. This review filled the gaps in the literature using the articles from PubMed and Google Scholar in order to fulfil obtaining free full-text articles that were publicly accessible. Keywords that were used included the following: "intravenous drug abuse", "complications of intravenous drug abuse", "bacterial infections in intravenous drug abuse", "complications of intravenous drug abuse" and "mental health implications of intravenous drug abuse". Filters applied include free full text, English language and articles between 2015 and 2023 in order to increase the validity of the studies used. The articles were curated in a Google document where all members of the study accessed and edited the information. A basic outline was made, and this paper was developed over Zoom meetings.

Implications of comorbidities

Psychiatric Comorbidities

Certain comorbidities have an already elevated risk of bacterial infections on their own. For example, people with psychiatric illnesses under International Classification of Diseases (ICD) codes F20-29 (schizophrenia, schizophreniform, and other non-mood psychotic disorders) were found to be at a higher risk of staphylococcal infections [23]. However, in one study looking at bacteremia, PWID who had psychiatric comorbidities had a significantly reduced risk of developing the infection, possibly due to receiving vigilant care as part of their psychiatric treatment [21]. PWID with alcohol use disorder are also a vulnerable group in terms of bacterial infections. Heavy alcohol consumption has been known to suppress the immune system making the host susceptible to diseases like tuberculosis and *Streptococcus pneumoniae* infection [24,25].

Cardiac Comorbidities

The comorbid cardiac conditions associated with severe bacterial infection among IVDUs are hypertension, thrombosis (deep vein thrombosis, pulmonary embolism), congestive heart failure, and valvular conditions [9]. *S. aureus* is the most common bacterial infection leading to infective endocarditis [26]. Prolonged hospitalization due to cardiac comorbidities increases nosocomial infection among IVDUs. Prolonged immobilization among IVDUs due to impairment in consciousness increases the risk of DVT and pressure ulcers, leading to infections [27]. Underlying local and distant site tissue damage helps create a favorable environment for the adherence and proliferation of bacteria [9]. The valvular conditions with damaged valves or prosthetic valves are prone to infections with *Staphylococcus epidermidis*, viridans streptococci, and *Streptococcus bovis* due to turbulence-mediated endothelial damage [9]. *S. aureus* prosthetic valve endocarditis (PVE) is among the most morbid bacterial infections. Early PVE infection is assumed to be caused by accidental seeding during surgery or due to bloodstream dissemination in the first hours to months postoperatively. Fibronectin and fibrinogen coat these areas and are thought to be a possible nidus for infection [28].

Respiratory Comorbidities

Comorbid conditions like asthma and chronic obstructive pulmonary disease increase the risk of infection among IVDUs. Pneumonia and tuberculosis have been found as the most common pulmonary complications [29]. Aspiration pneumonia and community-acquired pneumonia (CAP) are more frequent in people who inject drugs [29,30]. Aspiration pneumonia is increased among drug users due to impairment of consciousness (ranging from psychosis to coma), inhibition of cough, and alteration in gag reflexes [29,30]. In IVDUs, infectious pneumonia can be caused by bacteria and viruses. Commonly isolated bacterial pneumonia pathogens include gram-positive cocci, gram-negative rods, and anaerobic bacteria in aspiration pneumonia [29]. CAP is also seen among drug abusers. Intravenous drug use has additionally been related to greater severity of CAP, which might be evident by the development of empyema, complicated parapneumonic effusion, or the need for mechanical ventilation [31]. The most common bacterial infective organisms in IVDUs are *Staphylococcus aureus* and *Streptococcus pneumoniae* [29]. Infection with resistant bacteria like MRSA has been reported, leading to higher rates of intensive care admission due to greater pneumonia severity. Worldwide, IVDUs have higher TB infection prevalence and disease incidence compared to non-drug users. Additionally, patients with asthma exacerbation are observed to have many bacterial infections (*S. pneumoniae*, *Haemophilus influenzae*, and *Moraxella catarrhalis*) [32].

Diabetes Mellitus

IVDUs with diabetes mellitus (DM) have an increased risk of bacterial infection due to altered immune function [33]. A higher prevalence of infections in diabetics is caused by a hyperglycemic environment [34,35]. *Staphylococcus epidermidis* was the primary strain segregated from the skin of diabetic patients. *S. aureus* was the second most common [34]. Soft tissue infections like furuncles, folliculitis, and subcutaneous abscess are of particular concern in IVDUs with DM [33,34]. PWID with DM have an increased risk of osteomyelitis, sepsis, post-operative infections, and urinary tract infections. Patients with diabetes are frequently associated with the atypical infection, including streptococcal infection and Fournier’s gangrene [33]. IDU is commonly associated with noncompliance with insulin therapy and impacts the secretion of counter-regulatory hormones that promotes hyperglycemia [35].

Renal Comorbidities

Sepsis and bacterial infections are common in IVDUs with end-stage renal disease (ESRD). The immunocompromised condition of uremia, age, and diabetes, as well as the recurrent use of intravascular catheters in hemodialysis patients has been connected with an increased rate of infections [36]. There is a higher rate of MRSA infection in hemodialysis patients. The other causative organisms are *S. epidermidis* and coagulase-negative staphylococci (CNS), which are gram-positive organisms. Gram-negative organisms like *Escherichia coli*, *Enterobacter* species, and *Klebsiella* species are commonly seen in hemodialysis patients [37].

Strains of bacteria

Table 1 describes the most common bacterial strains identified in intravenous drug users from the studies included in this literature review. On comparing the bacteria causing infections among IVDUs, methicillin-resistant *S. aureus *had the highest incidence of causing infective endocarditis, most commonly involving left-sided valves and prosthetic valves, with HIV and liver disease being the common comorbidities. MRSA is also known to cause skin and soft tissue infections at the injection site. The main predictor of in-hospital mortality among PWID was *S. aureus* infection [38].

**Table 1:** Description of most common bacterial strains identified in intravenous drug users

Bacteria	Incidence	Underlying comorbidity (renal, cardiac, etc.)	Any relation with a specific disease?	Specific strains of bacteria	Regions studied
Staphylococcus	65.9%	HIV, prior infective endocarditis, liver disease	Infective endocarditis, cellulitis	*S. aureus* [38]	Europe, North America, Africa, Asia and South America
Serratia	36%	Hepatitis C	Osteomyelitis, endocarditis	*S. marcescens* [39]	North America (Ohio)
Streptococcus	26%	-	Skin and soft tissue abscess	*S. dysgalactiae* [40]	Europe (Switzerland)
Enterococcus	8.8%	-	Infective endocarditis	*E. faecalis* [41]	South Africa
Pseudomonas	7%	-	Infective endocarditis	*P. aeruginosa* [22]	North America

*Serratia marcescens* has been known to cause osteomyelitis and endocarditis among intravenous drug users, with a 36% incidence, with hepatitis C virus (HCV) infection being a comorbidity [39]. The manifestations of *Streptococcus dysgalactiae* in intravenous drug users include skin and soft tissue infections, with an incidence of 26%, varying from a single episode to multiple episodes [40]. *Enterococcus faecalis* is another bacteria causing infective endocarditis, with 8.8% incidence, with severe right ventricular dysfunction and pulmonary hypertension [41].

According to historical trends, PWID are considered to be at the highest risk of infection with gram-negative bacilli like *P. aeruginosa*. Subsequently, empirical antibiotic therapy, including anti-pseudomonal coverage received by patients, has been shown to reduce the incidence to 7% [22]. Most of the studies conducted were within the regions of North America that may be a misrepresentation of global data, as, firstly, underreporting of drug abuse may be a confounding factor and there are limitations in generalizing the aforementioned results to a global population.

Antibiotic susceptibility

Among many bacteria causing blood, skin and soft tissue infections, *Staphylococcus aureus*, *Streptococcus* and *Pseudomonas* are found to be most prevalent [42,43]. Among bacterial skin infections, cellulitis is common in IVDUs [44]. MRSA is one of the prevalent organisms in patients with a history of IV drug use. It should be treated by initiating trimethoprim/sulfamethoxazole 800 mg/160 mg twice a day for five days, along with cephalexin 500 mg every six hours. Clindamycin 300-450 mg every six hours is recommended for patients allergic to trimethoprim-sulfamethoxazole. If no improvement is observed in patients after 48 hours of initiating antibiotic therapy, antibiotics can be considered given for a longer duration [45]. Hospitalization may be required for patients who do not respond to outpatient treatment, are immunocompromised, or have systemic signs of infection. In such cases, IV antibiotics should be started to cover against group A strep. Therapy should be initiated with vancomycin, with subsequent de-escalation to trimethoprim/sulfamethoxazole, when the patient is stable. Broad-spectrum antibiotic coverage with vancomycin and piperacillin-tazobactam or a carbapenem is essential for immunocompromised patients requiring hospitalization [45].

A more severe type of skin infection that can also be found in IVDUs is necrotizing fasciitis. It is often caused by group A β-hemolytic streptococcus (GAS) and occasionally *S. aureus*. It is associated with a more than 30% mortality rate. Both medical and surgical treatment is required to treat necrotizing fasciitis. Aggressive antibiotic therapy should be initiated as soon as possible while the patient awaits surgical evaluation and treatment. MRSA-active antibiotics such as vancomycin, daptomycin, linezolid, or ceftaroline are required for such patients. In addition, a broad-spectrum agent, such as piperacillin-tazobactam, ampicillin-sulbactam, ticarcillin-clavulanate, extended-spectrum cephalosporins or carbapenems, against gram-negative pathogens is also given. Metronidazole or clindamycin should be added if the selected regimen lacks anaerobic activity [46].

Some skeletal infections, including septic arthritis and osteomyelitis, can also be caused by IV drug use. Among such patients, MRSA and *Pseudomonas* are the most common microbes causing these infections [47]. Therefore, for *Pseudomonas* treatment, beta-lactam antibiotic regimens are preferred, including ceftolozane-tazobactam, ceftazidime-avibactam, meropenem, ceftazidime or piperacillin-tazobactam with amikacin or colistin depending on local epidemiology and susceptibility of prior isolates [48].

Among the pathogens causing infective endocarditis, *S. aureus* is the most prevalent bacteria found preoperatively in blood cultures [42]. Approximately 26.6% of the cases of IE are caused by *S. aureus*. This organism is the most common cause of right-sided IE in intravenous drug users. IE is related to significantly high morbidity and mortality rates. Therefore, early diagnosis and appropriate treatment are required to achieve a cure. The inpatient mortality rate for it is approximately 30% [43]. To lower the mortality, the presence of complications of IE, i.e., septic shock, brain hemorrhage, renal failure or heart failure, echocardiography findings, and other prognostic factors should be considered while crafting a management plan and choosing between surgical intervention and medication. Similarly, selecting the correct antibiotic and appropriate duration of administration is essential to obtain good patient outcomes. The treatment recommended for methicillin-sensitive *S. aureus* (MSSA) in patients with native valves is flucloxacillin 2 g taken every four to six hours. For patients with a prosthetic valve, a combination of three antibiotics is recommended: flucloxacillin 2 g, every four to six hours; rifampicin 450-600 mg, twice a day and gentamicin 1 mg/kg, twice daily (later for two to six weeks). In patients with native valves, vancomycin with the weight-based dose is recommended for MRSA. In patients with a prosthetic valve, vancomycin (weight-based dose), with rifampicin 450-600 mg twice daily, is recommended twice daily, and gentamicin 1 mg/kg twice daily (later for two to six weeks) [43,49].

Another organism responsible for causing IE in IVDUs is *Enterococcus faecalis*. Monotherapy with beta-lactam antibiotics does not prove to provide any bactericidal activity against systemic infections. However, double beta-lactam therapy with ampicillin plus ceftriaxone or ampicillin plus gentamicin for 14 days has been proven beneficial in treating systemic infections [50]. *Serratia marcescens* can also cause infective endocarditis and osteomyelitis in IVDUs. A combination of beta-lactam with either a fluoroquinolone or an aminoglycoside is used for treatment [51].

Besides endocarditis, injection site contamination in IVDUs can cause the hematogenous spread of bacteria and lead to bloodstream infection (BSI) and sepsis [49]. Therefore, treatment in patients with suspected new BSI should include an antibiotic with activity against *Pseudomonas* species and *Stenotrophomonas maltophilia*, the two most common gram-negative isolates. This may include levofloxacin or an antipseudomonal carbapenem with trimethoprim-sulfamethoxazole. In addition, vancomycin can be added to this treatment plan if it is not already part of the infective endocarditis regimen to cover *Enterococcus *species [10]. Table 2 summarizes the antibiotic susceptibility of different micro-organisms and their related systemic manifestations.

**Table 2:** Summary of antibiotic susceptibility to the different pathological micro-organisms identified in intravenous drug abusers MRSA, methicillin-resistant *Staphylococcus aureus*; MSSA, methicillin-sensitive *S. aureus*

Type of infection	Common bacterial strains	Treatment options
Skin infections (cellulitis, necrotizing fasciitis)	MRSA, group A β-hemolytic streptococcus (GAS)	Trimethoprim-sulfamethoxazole 800 mg/160 mg twice a day for five days with cephalexin 500 mg every 6 hours; clindamycin 300-450 mg every 6 hours is recommended for patients allergic to trimethoprim-sulfamethoxazole. If no improvement is observed in patients after 48 hours of initiating antibiotic therapy, antibiotics can be considered for a longer duration [45]. Another consideration is that MRSA-active antibiotics such as vancomycin, daptomycin, linezolid, or ceftaroline are required for such patients. A broad-spectrum agent against gram-negative pathogens, such as piperacillin-tazobactam, ampicillin-sulbactam, ticarcillin-clavulanate, extended-spectrum cephalosporins or carbapenems, is also given. Metronidazole or clindamycin should be added if the selected regimen lacks anaerobic activity [46].
Skeletal infections (septic arthritis and osteomyelitis)	MRSA and *Pseudomonas* [47]	For *Pseudomonas* treatment, beta-lactam antibiotic regimens are preferred, including ceftolozane-tazobactam, ceftazidime-avibactam, meropenem, ceftazidime or piperacillin-tazobactam with amikacin or colistin [48].
Infective endocarditis	MSSA, *Enterococcus faecalis*, *Serratia marcescens*	In patients with native valves, flucloxacillin 2 g every 4-6 hours is recommended. In patients with a prosthetic valve, a combination of 3 antibiotics is recommended: flucloxacillin 2 g, every 4-6 hours, rifampicin 450-600 mg, twice a day, and gentamicin 1 mg/kg, twice daily (later for 2-6 weeks). In patients with a prosthetic valve, vancomycin (weight-based dose), with rifampicin 450-600 mg twice daily, is recommended twice daily, and gentamicin 1 mg/kg twice daily (later for 2-6 weeks) [43,49]. Double beta-lactam therapy with ampicillin plus ceftriaxone or ampicillin plus gentamicin for 14 days has proven beneficial in treating systemic infections [50]. Beta-lactam with either a fluoroquinolone or an aminoglycoside is used for treatment [51].
Bloodstream infection (sepsis)	*Pseudomonas* species and *S. maltophilia*	Levofloxacin or an antipseudomonal carbapenem with trimethoprim-sulfamethoxazole [10]

Discussion

There is a significant prevalence of IDU across the globe. A total of 179 countries worldwide have exhibited IDU in the age bracket of 15-64. As reported by the 2017 systematic review [1], IDU has a detrimental effect on almost all organ systems, which may be communicable or non-communicable [17]. PWID are susceptible to various infections, including viral and invasive bacterial infections, with skin and soft tissue infections being the chief reason for hospitalization in this group [8,17]. This is formidable in view of public health as hospitalizations for infections related to IDU are distinguished by a prolonged stay at the hospital as well as frequent readmission rates, higher patient-directed discharge, and increased intermediate to long-term mortality leading to significant expense to the healthcare system [8][9]. Furthermore, the increased need for antibiotics in PWID contributes to the development of multidrug-resistant bacteria and MRSA colonization [6,11,15]. Infected PWIDs can eventually introduce drug-resistant strains to the general public or immunocompromised individuals [11].

Through our review, we found that comorbidities compound susceptibility to infections in PWID. For instance, cardiac comorbidities, including hypertension, thrombosis, congestive heart failures, and valvular conditions (prosthetic or damaged heart valves) further predispose PWID to infection [9]. Similarly, heavy alcohol consumption exacerbates the already susceptible PWID to infection as it can undermine the innate and adaptive immune systems [24,25]. Bacterial trends most commonly infecting PWID have remained stable over the years, with no significant change being seen. The most prevalent bacteria seen infecting PWID include *Staphylococcus aureus* along with MRSA, which commonly causes infective endocarditis among other skin and soft tissue infections. Comorbidities such as prosthetic valve endocarditis and liver disease result in a higher incidence of infection [38]. Similarly, *Serratia marcescens* causes osteomyelitis and endocarditis in PWID [39]. *Streptococcus dysgalactiae* leads to skin and soft tissue infections varying from a single episode to multiple episodes [40]. *Enterococcus faecalis* is another bacteria causing infective endocarditis and can result in severe right ventricular dysfunction and pulmonary hypertension [41]. According to historical trends, PWID are vulnerable to infection with gram-negative bacilli like *Pseudomonas aeruginosa* [22]. Conclusively, preexisting comorbidities are an aggravating factor for infection in this already vulnerable population.

However, it is important to consider the limitations present in our study. We have identified that the demographics play a significant role in assessing bacterial infection trends, but due to unavailability of resources, it may not be feasible to obtain accurate reports of intravenous drug abuse in countries where a system to accurately document the incidence of intravenous drug abuse may not be present. It is also important to note that antibiotic guidelines in different hospitals within the same state or city may be different due to different antibiograms. The prevalence of infections, such as MRSA, may have different incidences in different localities leading to the usage of different first-line antibiotics, even for empiric coverage. Therefore, though our review is able to establish a general rule of thumb for appropriate antibiotic usage for the organisms present, it is essential to review the local antibiogram to establish the most accurate antibiotic usage to avoid increase in antibiotic resistance or ineffectiveness in treating a patient's condition.

Keeping in view that PWID are prone to infection and bacterial trends have remained stable, optimum healthcare must be available in a timely manner. An appropriate antibiotic regimen remains the key to the management of IDU-associated infections. However, excessive antibiotic use may be detrimental. Most PWID admitted to the hospital for IDU-related infection are administered empirical antipseudomonal therapy, which may be needless as *Pseudomonas* remains an uncommon cause of infection [22]. Empirical antimicrobial therapy should be focused on the most common organisms involved in IDU-associated infections, such as gram-positive *Staphylococcus *and *Streptococcus* [22]. Additionally, limited data is available on oral antibiotic use for treating bacteremia in PWID and intravenous antibiotics remain the mainstay of treatment. However, venous access may be difficult in some patients, requiring the retention of catheters and thereby increasing the rate of catheter-associated complications. Furthermore, central venous catheters can be exploited by PWID to inject recreational drugs [7]. Therefore, oral treatment substitutes should be explored. Moreover, harm reduction measures have helped decrease infection in PWID; these include education and counseling in hygienic practices and training in safe injection techniques. Interventions such as opioid substitution therapy and supervised injecting facilities can assist in lowering infections in PWID. Further research in optimum antibiotic regimens can help decrease the burden of disease in PWID.

## Conclusions

Although the bacterial trends have remained stable over the years, the presence of comorbidities in PWID makes them more susceptible to severe forms of infections. For instance, underlying hypertension or valvular conditions further predispose patients to infections. Common infections in PWID include cellulitis, joint infections, infective endocarditis and sepsis. Although current antibiotic guidelines have been adequate in treating infections within PWID, it is imperative to note the use of appropriate region-based antibiograms to determine the best use of antibiotics to avoid contribution towards antibiotic resistance. Furthermore, considering the risks associated with intravenous therapy and limited clinical data, there is a critical need to investigate the optimum antibiotic regimen and dosages to suppress the emergence of resistance, especially in PWID with comorbidities that may influence the choice of antibiotics.
